# Effects of Supplementing *Stevia rebaudiana* Bertoni Extract in the Diet of Yellow-Feathered Broilers on Meat Quality

**DOI:** 10.3390/ani16071026

**Published:** 2026-03-27

**Authors:** Xiao Peng, Xin Gao, Jing Wang, Zhen Li, Yunfei Song, Jianguo Zeng, Wei Xiang, Xiubin Liu

**Affiliations:** 1 College of Animal Science and Technology, Hunan Agricultural University, Changsha 410128, China; 2 Chinese Medicinal Materials Breeding Innovation Center, Yuelushan Laboratory, Changsha 410128, China; 3 Guilin Layn Natural Ingredients Corporation, Guilin 541000, China; 4 College of Veterinary Medicine, Hunan Agricultural University, Changsha 410128, China; 5 College of Horticulture, Hunan Agricultural University, Changsha 410128, China

**Keywords:** *Stevia rebaudiana* Bertoni extract, phytogenic feed additives, yellow-feathered broiler, meat sensory traits, oxidative stability, TVB–N

## Abstract

Improving meat quality using safe phytogenic feed additives is of practical interest in yellow-feathered broiler production. This study aimed to evaluate the effects of dietary *Stevia rebaudiana* Bertoni extract (SBE) supplementation on meat quality traits. A total of 510 one-day-old, female yellow-feathered broilers were randomly allocated to six dietary treatments (0, 50, 100, 200, 300, and 400 mg/kg SBE) for 63 days. Meat sensory attributes, routine physicochemical characteristics, amino acid and fatty acid composition, electronic-nose profiles, and total volatile basic nitrogen (TVB–N) during refrigerated storage were assessed. Overall, the results provide evidence that dietary SBE supplementation is associated with changes in multiple meat quality-related parameters, supporting further evaluation of SBE as a phytogenic feed additive in yellow-feathered broilers.

## Simple Summary

Yellow-feathered broilers are widely consumed in China, and the industry needs safe dietary strategies to improve meat quality and maintain freshness under reduced antibiotic use. Stevia rebaudiana Bertoni extract (SBE) is a phytogenic ingredient containing bioactive compounds with potential antioxidant activity, but its effects on broiler meat quality are not well established. Therefore, this study evaluated different dietary SBE inclusion levels in yellow-feathered broilers and assessed sensory attributes, physicochemical quality, and freshness-related indicators during refrigerated storage. The findings support SBE as a practical phytogenic feed additive candidate to enhance meat quality and help delay quality deterioration during cold storage.

## 1. Introduction

Poultry meat represents a significant portion of global meat consumption, with China standing out as both a major producer and consumer of poultry products. The increasing consumer demand for food safety and high-quality meat, coupled with concerns regarding antibiotic residues, has driven the development of phytogenic feed additives as safe and environmentally friendly alternatives to enhance production efficiency and meat quality. These plant-derived extracts and compounds are rich in bioactive components, are widely regarded as safe, effective, environmentally friendly, and are readily available. These additives are rich in bioactive compounds, such as polyphenols, flavonoids, alkaloids, and essential oils, which have been reported to regulate physiological functions, enhance antioxidant capacity, and promote growth and development [1,2,3].

In China, yellow-feathered broilers represent a distinctive and commercially important poultry type and are widely valued for their sensory characteristics in traditional culinary uses. Compared with intensively selected white-feathered broilers, yellow-feathered broilers generally exhibit a slower-growing genetic background and differentiated metabolic patterns related to lipid deposition and muscle development, which are associated with consumer-valued meat quality traits (e.g., flavor, texture, and juiciness) [4,5]. Despite strong market demand, systematic evidence regarding phytogenic strategies to optimize meat quality in yellow-feathered broilers remains limited.

*Stevia rebaudiana* Bertoni is a perennial herb indigenous to subtropical regions of South America and is adapted to subtropical environmental conditions [6]. Stevia leaves contain steviol glycosides (e.g., stevioside and rebaudiosides), which are widely used as high-intensity, non-caloric sweeteners in the food and beverage industry [7]. Since its introduction into China in the 1970s and commercial cultivation starting in the 1980s, China has developed into one of the major producers of *S. rebaudiana* leaves and related extracts, supporting the availability of stevia-derived products for broader applications [8]. *Stevia rebaudiana* Bertoni extract (SBE) has attracted extensive research attention in recent years due to its antioxidant, anti-inflammatory, and antibacterial activities [9,10,11]. Dietary supplementation with SBE has been demonstrated to improve growth performance, antioxidant capacity, immune function, and intestinal health in yellow-feathered broilers [12]. Furthermore, *stevia*-residue extract has been shown to improve production performance and antioxidant capacity in growing-fattening pigs [13]. However, the dose-dependent effects of SBE on meat quality traits in yellow-feathered broilers have not been fully characterized.

Therefore, the present study aims to evaluate the effects of dietary SBE supplementation on meat-quality parameters in yellow-feathered broilers. Based on the antioxidant and metabolic regulatory roles of SBE bioactive compounds and the distinctive physiological background of yellow-feathered broilers, we hypothesized that dietary SBE supplementation would improve the meat quality of yellow-feathered broilers—including tenderness, color stability, amino acid composition, and shelf-life related indicators—and that these effects would vary according to the supplementation level.

## 2. Materials and Methods

### 2.1. SBE Preparation

**Figure 1:**
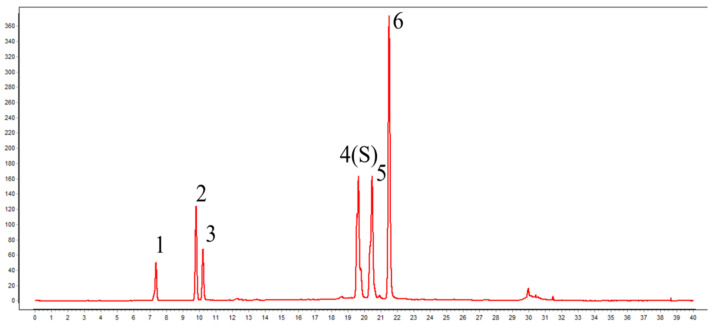
Characteristic atlas of *Stevia rebaudiana* extract. Peak 1: Neochlorogenic acid; Peak 2: Chlorogenic acid; Peak 3: Cryptochlorogenic acid; Peak 4: Iso Chlorogenic acid B; Peak 5: Iso Chlorogenic acid A; Peak 6: Iso Chlorogenic acid C.

The SBE utilized in this study was provided as a powder by Rhine Biotechnology Co., Ltd., (Guilin, China) containing ≥50% total chlorogenic acids, including chlorogenic acid, neochlorogenic acid, cryptochlorogenic acid, isochlorogenic acid A, isochlorogenic acid B, and isochlorogenic acid C. For HPLC analysis, 0.025 g of SBE was accurately weighed into a 50 mL volumetric flask and extracted with 50% methanol (approximately 30 mL) by ultrasonication for 30 min (250 W, 40 kHz) until fully dissolved. After cooling to room temperature, the solution was brought to volume with 50% methanol, mixed well, and filtered through a 0.22 μm membrane prior to injection. The liquid chromatography conditions were as follows: Waters 2695 system (Waters, Milford, MA, USA) equipped with a diode array detector (DAD), mobile phase A was acetonitrile and mobile phase B was 0.1% formic acid in water. Separation was performed on a Zorbax C18 column at 30 °C with a flow rate of 1.0 mL/min. Detection was at 327 nm; injection volume was 10 μL. The elution gradient was as follows: At 0 min, 10% mobile phase A; at 7 min, 17% mobile phase A; at 10 min, 17% mobile phase A; at 15 min, 25% mobile phase A; at 16 min, 26.5% mobile phase A; at 21 min, 26.5% mobile phase A; at 25 min, 28.5% mobile phase A; at 30 min, 95% mobile phase A; at 35 min, 95% mobile phase A; at 35.10 min, 10% mobile phase A; and finally, at 40 min, 10% mobile phase A. The liquid chromatogram of the stevia extract is depicted in Figure 1.

### 2.2. Study Animals and Design

In this experiment, 510 one-day-old, female yellow-feathered broilers (initial body weight: 35.06 ± 0.36 g at day 0) were purchased from Hunan Jitai Agriculture and Animal Husbandry Co., Ltd. (Changsha, China) and were randomly allocated into six treatment groups using random numbers generated in Microsoft Excel. Each treatment consisted of five replicate pens with 17 chickens per pen. Chickens were offered the basal diet during an initial seven-day adaptation period. Dietary supplementation with SBE was initiated on day eight. SBE was incorporated into the basal diet by first premixing it with a small portion of the basal diet and then stepwise mixing it into the complete diet to ensure homogeneity. The treatment groups included a blank control group (Blank), fed a basal diet, along with experimental groups (SBE–50, SBE–100, SBE–200, SBE–300, SBE–400), that received supplementation at 50 mg/kg, 100 mg/kg, 200 mg/kg, 300 mg/kg, and 400 mg/kg, respectively, in addition to the basal diet. The sample size was determined with reference to general minimum animal number requirements for poultry experiments and finalized based on the practical feasibility of the study. The experimental diet was a corn–soybean meal basal diet, formulated in accordance with the National Research Council (NRC, 1994) and the Chinese agricultural industry standard, “Nutritional Requirements of Yellow-Feathered Broiler Chicks” (NY/T 33–2004) (see Table 1 for composition and nutrient levels) [14].

The experiment was conducted in the animal laboratory of Hunan Agricultural University over a duration of 63 days. Before the experiment, the chicken house was cleaned, and a three-tiered cage rearing system was adopted. Each cage measured 200 cm × 58 cm × 62 cm (length × width × height) and had a wire-mesh floor. Inside each cage, there was one internal feed trough running along the front and two nipple drinkers per cage. Prior to chick placement, the temperature inside the chicken house was raised to 32–35 °C; thereafter, the temperature was reduced by 2–3 °C every week until it was maintained at 20–22 °C. The relative humidity in the chicken house was kept at 50–60%, with 16 h of light provided daily. During the experiment, the chickens were given ad libitum access to feed and water. Routine immunization procedures were implemented, with the specific schedule as follows: At 5 days of age, the Newcastle Disease and Infectious Bronchitis combined live vaccine was administered to the chicken via eye dropping; at 10 days of age, the inactivated Newcastle Disease vaccine was injected subcutaneously; at 14 days of age, the live Infectious Bursal Disease vaccine was given via nasal dropping; at 20 days of age, the Newcastle Disease and Infectious Bronchitis combined live vaccine was administered via nasal dropping; and at 28 days of age, the live Infectious Bursal Disease vaccine was given through eye dropping.

### 2.3. Sample Collection

Before slaughter, the chickens were fasted overnight for 12 h with free access to water. To minimize selection bias, broilers for slaughter were chosen based on their proximity to the average body weight of each replicate pen. On the following morning, the chickens were killed by jugular vein exsanguination. From each carcass, the breast and thigh muscles were excised, and all visible external fat and connective tissue were carefully removed before analysis. Breast muscle samples intended for sensory evaluation were vacuum-packaged and stored at 4 °C in a refrigerator (CZ3456, Haier, Changsha, China), and sensory evaluation was conducted within 24 h after slaughter. Breast and thigh muscle samples were used to measure pH and color (L*, a*, b*) at 45 min and 24 h post-mortem, with carcasses and samples kept at 4 °C between these time points. Muscle stored at 4 °C was also used for the determination of water loss rate and shear force (measured within 24 h post-mortem), drip loss (after 24 h storage at 4 °C) and total volatile basic nitrogen (TVB–N). Samples intended for amino acid, fatty acid and electronic-nose analyses were wrapped in aluminum foil, vacuum packaged and stored at −80 °C in an ultra-low temperature freezer (MDF-682VE, Songyang Biotechnology Co., Ltd., Beijing, China) until measurement (hold for no more than two months).

### 2.4. Measurement Indicators and Methods

Unless otherwise stated, all physicochemical and meat-quality measurements, including pH, meat color, water loss rate, drip loss, storage loss, cooking loss, shear force, proximate composition (moisture, crude protein, crude fat and ash), amino acid composition, fatty acid composition, TVB–N and electronic-nose sensor responses, were conducted on samples from five chickens per dietary treatment (*n* = 5). For each chicken and each trait, three technical replicates were performed, and the mean of the three replicates was used for statistical analysis. Sensory evaluation was carried out by six panelists, as described in the sensory analysis subsection.

#### 2.4.1. Sensory Evaluation

The sensory evaluation of chicken samples was conducted in accordance with the Specification for the Sensory Evaluation of Meat and Meat Products (GB/T 22210–2008) and a published method [15,16]. Each panelist evaluated six samples. Sensory attributes were scored using a five-point intensity scale (higher scores indicating greater intensity). Breast muscle was cut into strips of approximately 2–3 cm in length and about 1 cm in width and height, placed in white porcelain bowls and covered with water at a meat-to-water ratio of 1:5 so that the meat was fully immersed. Water was brought to the boil, and then the meat samples were added and cooked in water maintained at 80–85 °C (close to boiling but without vigorous boiling) for 20 min, without any seasoning. After cooking, samples were served warm for evaluation (55 °C).

Sensory evaluation was performed by six trained assessors, who scored each dietary treatment for eight attributes: color, juiciness, texture, odor, umami, flavor, tenderness and taste. Evaluations were carried out individually and without discussion among panelists. To minimize carry-over effects between treatments, panelists consumed pieces of peeled cucumber and rinsed their mouths between samples to clean the palate. The detailed sensory scoring criteria are presented in Table 2.

#### 2.4.2. Electronic Nose (E-Nose) Measurement

The detection of volatile odor components in meat samples was conducted following the method established by Xu et al. [17] using an electronic nose system (PEN 3.0, Airsense, Schwerin, Germany). Meat samples were first minced using a meat grinder to obtain a homogeneous mince. For each sample, 2.0 g of minced meat was accurately weighed and placed into a 15 mL headspace vial, which was immediately capped and sealed. The sealed vials were then incubated in a water bath at 85 °C for 360 s, and during incubation they were vortex-mixed for 5 s every 180 s to promote the release and equilibration of volatile compounds in the headspace before e-nose measurement.

Sensor responses were expressed as G/G_0_. All measurements were performed at room temperature (no temperature setting was applied). The assay procedure involved a manual single-sample injection, with a carrier flow 150 mL/min; injection volume 100 μL at 2.5 μL/s. The measurement and washout times were both 120 s. Sensors used in PEN3 e-nose and their performance description is in Table 3.

#### 2.4.3. Routine Physicochemical Analysis

Moisture, crude fat, crude protein, and ash contents of meat samples were determined according to AOAC Official Methods: Moisture by oven-drying (AOAC 950.46), crude fat by Soxhlet extraction (AOAC 960.39), crude protein by the Kjeldahl method (AOAC 981.10, using a nitrogen-to-protein conversion factor of 6.25), and ash by muffle furnace incineration (AOAC 920.153). All results were reported as % (*w*/*w*). Briefly, minced and homogenized meat samples were dried in a forced-air oven at 100–105 °C to constant weight for moisture determination. Crude fat was extracted from dried samples in a Soxhlet apparatus using petroleum ether as the extraction solvent, and crude fat content was calculated gravimetrically. For crude protein determination, samples were digested with concentrated sulfuric acid in the presence of a catalyst mixture, followed by steam distillation and titration to determine the total nitrogen; crude protein was then calculated using a conversion factor of 6.25. Ash content was determined by incinerating samples in a muffle furnace at 550–600 °C to constant weight, and the mass fractions of each component were calculated based on the corresponding mass differences.

In accordance with the “Objective Evaluation Method for the Edible Quality of Meat” (NY/T 2793–2015) [14], the following indicators were measured: pH value, utilizing an insertable pH meter (205, TESTO AG, Titisee-Neustadt, Germany) to assess the pH of the right breast muscle and thigh muscle of the chicken at 45 min and 24 h post-slaughter; meat color, measured using a meat colorimeter (NR20XE, Sanenshi Technology Co., Ltd. Shenzhen, China) to determine the L* (lightness), a* (redness), and b* (yellowness) of the right chest and thigh muscles at the same time points; water loss rate (%) was measured using a water-holding capacity meter (RH–1000, Runhu Instrument Co., Ltd., Guangzhou, China). Cylindrical muscle samples (approximately 2.5 cm in diameter and 1 cm in thickness) were prepared and weighed (S1), wrapped in two layers of medical gauze, and placed between 18 layers of medium-speed qualitative filter paper above and below. The samples were then pressed at 35 kg for 5 min in the instrument. After removing the gauze and filter papers, the samples were reweighed (S2), and water loss (%) was calculated from the difference between the initial and final weights. For drip loss (%), muscle samples were trimmed into strips (2 × 2 × 1 cm). Each strip was hooked with fine wire and suspended inside an inverted plastic cup without contact between the meat and the cup wall. Samples were stored at 4 °C for 24 h, then gently blotted with filter paper and reweighed. Drip loss was expressed as the percentage weight loss during hanging. For cooking loss rate (%), approximately 30 g of muscle was placed individually in heat-stable plastic bags with the opening upwards, heated in an 80 °C water bath until the geometric center reached 70 °C and was maintained for 10 min, then cooled at room temperature for 30 min. After removing the exuded fluid, the samples were reweighed, and cooking loss was calculated as the percentage reduction in weight after cooking. Shear force was determined on cooked breast and thigh muscle strips (1 × 1 × 5 cm), cut along the direction of the muscle fibers and trimmed free of fascia and visible fat. Each strip was sheared perpendicular to the fibers three times using a tenderness meter, and the mean value was recorded as shear force (N). Storage loss was assessed using blocks (30 × 10 × 10 mm) cut along the muscle fibers, weighed (M1), vacuum packaged, and stored at 4 °C for 48 h. After storage, samples were gently blotted and reweighed (M2). Storage loss was expressed as the percentage weight lost during refrigerated storage.

#### 2.4.4. TVB–N Determination

For TVB–N determination, 100 g of breast muscle and 100 g of thigh muscle were collected from each chicken, homogenized, and equally divided into seven portions. Each portion was packed in an individual sealed bag and stored at 4 °C. TVB–N was measured at 0, 12, 24, 36, 48, 60 and 72 h of refrigerated storage, using one portion per chicken and time point (*n* = 5 per treatment at each time point). TVB–N was determined using the semi-automatic Kjeldahl method, in accordance with the National Standard for Food Safety for the Determination of Volatile Basic Nitrogen in Food (GB 5009.228–2016) [18] and expressed as mg/100 g. Meat samples were homogenized, and 10 g of each sample (weighed to 0.001 g accuracy) was placed into a distillation tube. Subsequently, 75 mL of water was added, and the mixture was shaken to ensure thorough dispersion, followed by maceration at room temperature for 30 min. The semi–automatic Kjeldahl nitrogen analyzer (QW-KDN, Qiwei Instrument Co., Ltd., Hangzhou, China) was operated according to the manufacturer’s instructions.

#### 2.4.5. Fatty Acid

Fatty acid content was determined using a gas chromatograph (6890N, Agilent, Poway, CA, USA). Meat samples were removed from −80 °C storage and freeze-dried in a laboratory freeze dryer until constant weight. The freeze-dried samples were then ground to a homogeneous powder using a pestle and mortar. About 0.5 g of samples were weighed and placed in a 50 mL centrifuge tube. Then 4 mL of a benzene–petroleum ether (1:1) mixed solvent was added, sealed, and extracted for 24 h.

Rapid methylation: After 24 h of extraction, 4 mL of potassium hydroxide–methanol solution (0.4 mol/L) was added, vortexed for 3 min, and left to stand for 30 min. After adding ultrapure water to separate the layers, the upper layer was collected, and an appropriate amount of anhydrous sodium sulfate was added to remove water from the sample. 200 μL of the sample for testing was taken and diluted with 800 μL hexane.

Chromatographic conditions: Column SP-2560 (100 m × 0.25 mm × 0.2 μm, Merck, Darmstadt, Germany); carrier gas: High-purity nitrogen, flow rate 0.8 mL/min; injection volume: 1 μL, split ratio 20:1; FID detector: Temperature 280 °C, hydrogen 30 mL/min, air 400 mL/min; column temperature: Initial temperature 140 °C held for 5 min, then increased at 3 °C/min to 220 °C, held for 40 min.

#### 2.4.6. Amino Acid

Freeze-dried breast or thigh samples were ground; approximately 0.3 g was transferred to a 20 mL digestion tube, 10 mL of six mol/L HCl was added, and the tube was sealed and hydrolyzed at 110 °C for 22 h. After cooling, the hydrolysate was opened, filtered, washed several times with deionized water, and brought to volume in a 25 mL volumetric flask. A 200 μL aliquot of filtrate was dried to remove residual six mol/L HCl, then dissolved with 1 mL of 0.01 N HCl, transferred to a 10 mL volumetric flask, and brought to volume with deionized water. Samples were centrifuged at 15,000 rpm for 15 min or filtered through a 0.22 μm polyethersulfone aqueous syringe filter for analysis. Detection was carried out using a high-performance liquid chromatography system (1260, Agilent, USA) equipped with a G1311B quaternary pump, G1329B autosampler, G1330B thermostat, and G1316A column oven, together with a diode-array detector. The chromatographic column was an ACQUITY UPLC BEH column (100 mm × 2.1 mm, 1.7 μm, Waters, Milford, MA, USA). Mobile phase A consisted of 45% methanol, 45% acetonitrile, and 10% water, and mobile phase B consisted of a 10 mM Na_2_HPO_4_ and 10 mM Na_2_B_4_O_7_ mixture, with the pH adjusted to 8.2 using concentrated hydrochloric acid. The gradient of the mobile phase was as follows: 0 min, 2% mobile phase A; 0.35 min, 2% mobile phase A; 13.40 min, 57% mobile phase A; 13.50 min, 100% mobile phase A; 17.70 min, 100% mobile phase A; 17.80 min, 2% mobile phase A; 22.00 min, 2% mobile phase A.

### 2.5. Statistics and Analysis of Data

Statistical analyses were performed using SPSS 27.0 (IBM Corp., Armonk, NY, USA). Meat color (L*, a*, b*), pH, shear force, water loss rate, drip loss, storage loss, proximate composition (moisture, crude protein, crude fat and ash), amino acid composition, fatty acid composition, TVB–N values, e-nose sensor responses and sensory evaluation scores were analyzed by one-way analysis of variance (ANOVA), with dietary treatment as the main effect. For each trait, the value used for analysis was the mean of three technical replicates per chicken, and the chicken served as the experimental unit (*n* = 5 chickens per treatment). When the ANOVA indicated significant differences among treatments (*p* < 0.05), Duncan’s multiple range test was used to separate means. Data are presented as means ± SEM. Graphical representations were generated using GraphPad Prism 9.0 (GraphPad Software, San Diego, CA, USA) and Origin 2019 (OriginLab Corp., Northampton, MA, USA). Principal component analysis (PCA) of the electronic-nose sensor response data was performed in Origin 2019 after mean-centering and scaling the variables to unit variance, and the resulting principal components were used to visualize overall differences among dietary treatments.

## 3. Results

### 3.1. Sensory Evaluation of Meat Quality of Yellow-Feathered Broilers at Different Doses of SBE

**Figure 2:**
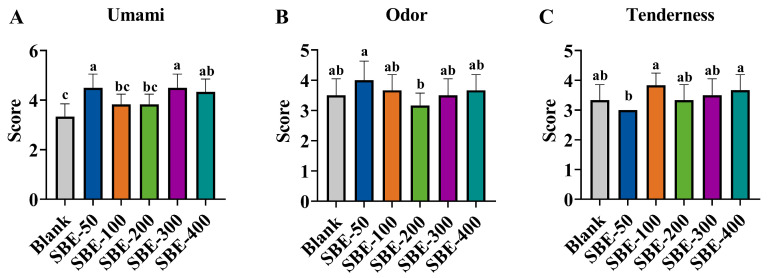
Selected sensory scores of different experimental groups. (**A**) Umami, (**B**) Odor, and (**C**) Tenderness (*n* = 6 panelists per treatment). Note: Different letters above the bars indicate significant differences among dietary treatments (*p* < 0.05). The complete sensory data for all evaluated attributes are presented in Supplementary Table S1.

For the sensory evaluation, only those attributes that showed significant differences among the experimental groups are presented in Figure 2. The complete dataset for all sensory attributes evaluated is provided in Supplementary Table S1. The results indicated that the umami score of the SBE–supplementation groups at 50, 300, and 400 mg/kg was significantly higher than that of the Blank group (*p* < 0.05; Figure 2A). The odor score of the SBE–50 group was significantly higher than that of the SBE–200 group (*p* < 0.05; Figure 2B), the tenderness score of the SBE–200 and SBE–400 groups was higher than that of the SBE–50 group (*p* < 0.05; Figure 2C), and the remaining attributes showed no significant differences (Table S1).

### 3.2. Study on the Effect of Different Doses of SBE on Volatile Components of Yellow-Feather Broilers Analyzed Based on E-Nose Technology

**Figure 3:**
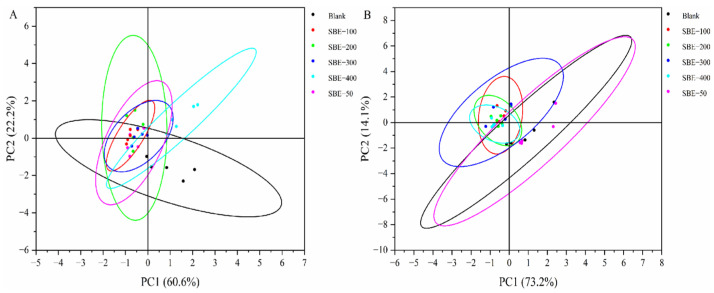
PCA diagram of G/G_0_ values of breast muscle samples from different experimental groups (**A**), PCA diagram of G/G_0_ values of thigh muscle samples from different experimental groups (**B**) (*n* = 5). The colored lines displayed in the PCA plot represent the 95% confidence ellipses for each corresponding group.

PCA of chicken breast meat (Figure 3A) showed that PC1 and PC2 together explained 82.8% of the variance (PC1, 60.6%; PC2, 22.2%), indicating that the model effectively captured the flavor differences between samples. PCA of thigh meat (Figure 3B) showed that PC1 and PC2 cumulatively explained 87.3% of the variance (PC1, 73.2%; PC2, 14.1%), indicating that the model effectively captured the flavor differences in thigh meat.

**Figure 4:**
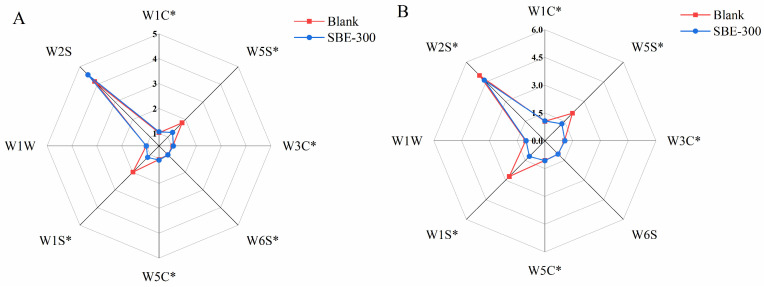
Radar plots of electronic-nose sensor response patterns (G/G_0_) for breast (**A**) and thigh (**B**) meat in the Blank and SBE–300 groups (*n* = 5). Note: * indicates a statistically significant difference (*p* < 0.05). Complete sensor response data for all dietary treatments are presented in Supplementary Table S2.

Figure 4 presents radar plots of electronic-nose sensor response patterns (G/G_0_) for breast (Figure 4A) and thigh (Figure 4B) meat, comparing the Blank and SBE–300 groups. The complete sensor response data for all dietary treatments are summarized in Supplementary Table S2. For breast meat (Figure 4A, Table S2), W1C and W3C responses in SBE–50 to SBE–300 were significantly higher than in the Blank and SBE–400 groups (*p* < 0.05), whereas W1S and W5S responses in SBE–50 to SBE–300 were significantly lower than in the Blank and SBE–400 groups (*p* < 0.05). In SBE–100 to SBE–400 groups, W1S and W5S responses were significantly lower than in the Blank group (*p* < 0.05). SBE–100 to SBE–400 groups had significantly higher W6S and W5C responses than the Blank group (*p* < 0.05). In addition, the SBE–200 and SBE–400 groups had significantly higher responses for W2S than the Blank group (*p* < 0.05), and the SBE–400 group had significantly higher responses for W1W than the other SBE–supplementation groups (*p* < 0.05). For thigh meat (Figure 4B, Table S2), W1C, W3C, and W5C responses were significantly higher in SBE–100 to SBE–400 than in SBE–50 and the Blank group (*p* < 0.05); W5S responses were significantly higher in SBE–200, SBE–300, and SBE–400 (*p* < 0.05), whereas W1S responses were significantly lower than in SBE–50 and the Blank group (*p* < 0.05).

### 3.3. Study on the Effect of Different Doses of SBE on Routine Physicochemical Analysis Indexes of Meat Quality of Yellow-Feather Broilers

For breast meat, crude fat was significantly higher in all SBE–supplementation groups, except the SBE–200 group than Blank group (*p* < 0.05, Table 4). The a* values were significantly higher and L*_45min_ values were significantly lower in the SBE–300 and SBE–400 groups compared with the Blank group (*p* < 0.05, Table 4). The a* values were significantly higher in the SBE–400 group. The b* values were significantly higher in all SBE–supplementation groups except the SBE–50 group than in the Blank group (*p* < 0.05, Table 4). In addition, L* values were significantly higher in all SBE–treated groups than in the Blank group (*p* < 0.05, Table 4). The pH values measured at 45 min and 24 h post-slaughter were higher in all SBE–supplementation groups than in the Blank group, except for the SBE–50 group (*p* < 0.05, Table 4). Shear force was significantly lower in all SBE–supplementation groups compared to the Blank group (*p* < 0.05, Table 4), and drip loss was significantly lower in the SBE–200 to SBE–400 groups than in the Blank group (*p* < 0.05, Table 4). The results of thigh meat showed significant differences in crude fat, moisture, crude protein, and crude ash contents between the treatment groups and the Blank group (*p* < 0.05, Table 4). The L* values of all SBE–supplementation groups were significantly lower than those of Blank group, except for SBE–50 group, which was significantly higher than that of Blank group in each SBE–supplementation group (*p* < 0.05, Table 5). Except for the SBE–50 group, pH values were higher in all SBE–supplementation groups than in the Blank group (*p* < 0.05, Table 5). Drip loss was significantly higher in all SBE–supplementation groups than in Blank group (*p* < 0.05, Table 5), and the rest of the measurements did not detect significant differences between the groups (*p* > 0.05, Table 5).

### 3.4. Study on the Effect of Different Doses of SBE on the Changes in TVB–N Content in Yellow-Feather Broilers Under 4 °C Storage Condition

**Figure 5:**
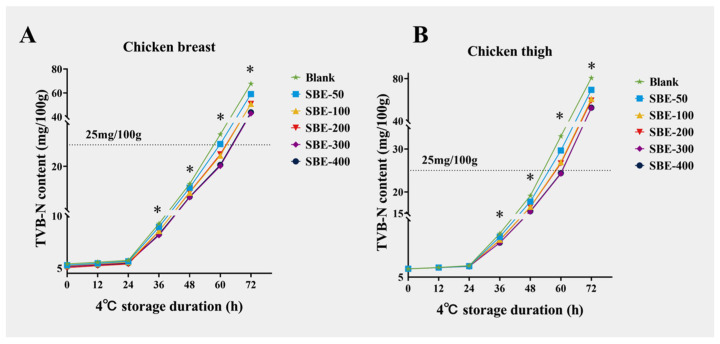
Changes in volatile basic nitrogen (TVB–N) content in chicken breast at different time periods stored at 4 °C (**A**), changes in volatile basic nitrogen (TVB–N) content in chicken thigh at different time periods stored at 4 °C (**B**), mg/100 g (*n* = 5). Note: * indicates a statistically significant difference (*p* < 0.05).

Figure 5 shows that the TVB–N content of all the groups of samples gradually increased with storage time. Across storage times, TVB–N in the SBE–300 and SBE–400 groups was consistently lower than that of the other groups at all time points. Notably, after 60 h of storage, TVB–N in SBE–300 and SBE–400 was significantly lower than in the Blank group and remained below 25 mg/100 g (*p* < 0.05).

### 3.5. Effects of Different Doses of SBE on Fatty Acid Composition of Yellow-Feathered Broiler Chicken

**Figure 6:**
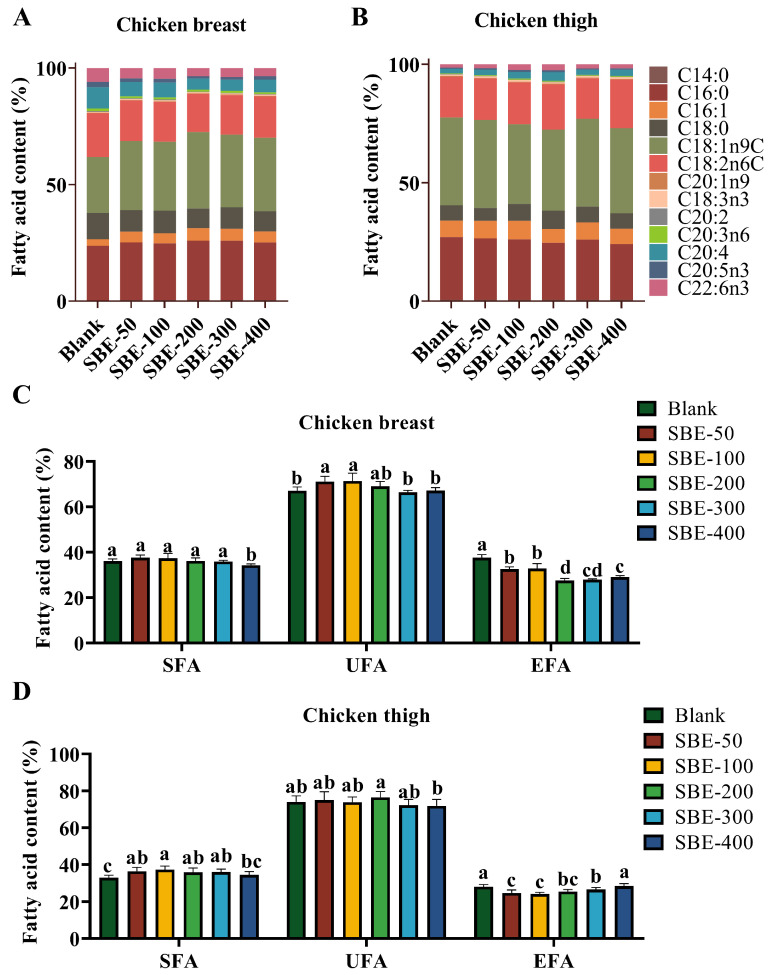
Fatty acid proportions in chicken of different test groups. Proportions of various fatty acids (**A**), UFA, SFA and EFA (**C**) in chicken breast. Proportions of various fatty acids (**B**), UFA, SFA and EFA (**D**) in chicken thigh (*n* = 5). Note: Different letters above the bars indicate significant differences among dietary treatments (*p* < 0.05).

In both breast and thigh, C18:1n9C, C16:0, and C18:2n6C were higher in the fatty acid composition of chicken breasts and thighs (Figure 6A,B). All SBE–supplementation groups had significantly lower essential fatty acids (EFA) in breast meat than the Blank group (*p* < 0.05, Figure 6C). In thigh muscle, all SBE–supplementation groups except SBE–400 had a significantly higher proportion of unsaturated fatty acid (UFA) and a significantly lower proportion of EFA in the thigh muscle than the Blank group (*p* < 0.05, Figure 6D).

### 3.6. Study on the Effect of Different Doses of SBE on the Amino Acid Composition of Yellow-Feather Broilers

**Figure 7:**
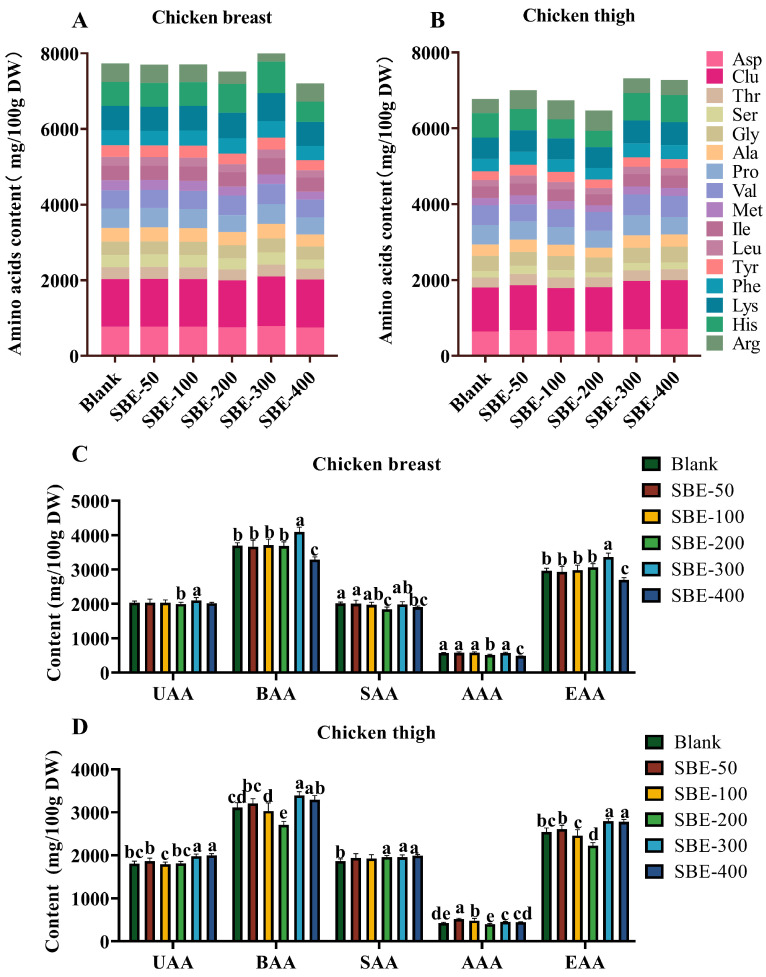
Amino acid contents in chicken muscle of different test groups. Contents of various amino acids (**A**), UAA, BAA, SAA, AAA and EAA (**C**) in chicken breast. Contents of various amino acids (**B**), UAA, BAA, SAA, AAA and EAA (**D**) in chicken thigh (*n* = 5). Note: UAA = Asp + Glu; SAA = Thr + Ser + Gly + Ala + Pro; BAA = Val + Met + Ile + Leu + Tyr + Phe + Lys + His + Arg; AAA = Tyr + Phe; EAA = Thr + Lys + Leu + Ile + Met + Phe + Val. Note: Different letters above the bars indicate significant differences among dietary treatments (*p* < 0.05).

Figure 7 shows the contents of 16 amino acids in chicken meat. Figure 7A,B depicts the distribution of various amino acids in chicken breast and thigh meat, respectively, and the results indicated that there were differences in the amino acid composition between the different experimental groups (Blank group, SBE–50 to SBE–400) (*p* < 0.05). Among the amino acids in chicken breast meat, the concentrations of bitter amino acids (BAA) and essential amino acids (EAA) were significantly higher in the SBE–300 group compared with the Blank group. On the contrary, BAA, sweet amino acids (SAA), aromatic amino acid (AAA), and EAA concentrations were significantly lower in the SBE–400 group than in the Blank group (*p* < 0.05, Figure 7C). In addition, in chicken thigh meat, umami amino acids (UAA), BAA, SAA, and EAA concentrations were significantly higher in both the SBE–300 and SBE–400 groups relative to the Blank group (*p* < 0.05, Figure 7D).

## 4. Discussion

Our previous work showed that dietary SBE improved the growth performance, antioxidant capacity, immunity, and intestinal health of yellow-feathered broilers [12]. In that growth-performance trial, supplementation at 100–200 mg/kg significantly increased final body weight and average daily gain and improved feed-to-gain ratio, whereas 300 mg/kg tended to depress growth performance; therefore, 100–200 mg/kg was recommended as the appropriate supplementation range under routine production conditions. Building on this physiological and production background, the present study focuses specifically on post-mortem meat quality traits in yellow-feathered broilers in order to clarify how different SBE inclusion levels modulate tenderness, color, flavor-related amino acids, and shelf-life indicators.

Sensory characteristics are crucial for evaluating flavor quality and consumer acceptance. While consumers primarily select meat based on color, texture, flavor, and preference, traditional sensory evaluation is time-consuming and subjective. The e-nose offers an objective, rapid, sensitive, and efficient alternative for characterizing food aroma by mimicking human olfaction [19,20]. The results of this study indicated that dietary SBE supplementation positively influenced the sensory flavor attributes of chicken meat, particularly enhancing broth freshness and tenderness. Sensory scores revealed that the fresh flavor intensity scores for the SBE–50, SBE–300, and SBE–400 groups were significantly higher than those of the Blank group (*p* < 0.05), consistent with previous studies [21]. E-nose analysis demonstrated that the responses of the W1S and W5S were significantly lower, suggesting a reduction in the relative abundance of methyl analogs and nitrogen oxides. Since methyl analogs and nitrogen oxides are often associated with spoilage metabolites, their reduction suggests fresher flavors and fewer undesirable odors. This effect was particularly pronounced in the SBE–100 to SBE–300 range, indicating that medium to high doses of SBE are more effective in modulating volatile flavor compounds.

Flesh color is a crucial sensory attribute that significantly influences consumer purchasing decisions. It can be objectively quantified using the L*, a* and b* parameters in the CIELAB color space [22]. The a* value is primarily affected by myoglobin (Mb) [23], as after slaughter, Mb binds to O2 in the air to form bright red MbO2, which subsequently oxidizes over time to high iron myoglobin (brown) [24,25]. Polyphenol-rich plant extracts have been demonstrated to stabilize and enhance the a* value of meat products [26]. In the present study, the a* value of chicken breast meat in the SBE–300 group was significantly higher than that of the Blank group. This enhancement may be attributed to SBE’s ability to scavenge lipid peroxidation products, thereby protecting Mb from oxidative denaturation and maintaining a higher percentage of the desired MbO_2_ state. Tenderness is inversely related to shear force; reactive oxygen species (ROS) promote cross-linking of myofibrillar proteins (e.g., actin, myosin), thereby reducing tenderness. Antioxidants improve tenderness by scavenging ROS and preserving myofibril integrity [27]. In this study, breast muscle shear was significantly reduced in the SBE–300 group (*p* < 0.05). Additionally, pH is typically associated with improved tenderness and juiciness [28,29]; however, excessively high pH (>6.1) may lead to undesirable flavor development [29]. Mechanistically, steviol glycosides bind to sweet taste receptors (T1R2–T1R3), triggering endoplasmic reticulum Ca^2+^ release and the activation of TRPM5 channels. Elevated intracellular Ca^2+^ levels form the Ca^2+^/calmodulin complex, which activates CaMKKβ, subsequently phosphorylating the α–subunit of AMP–activated protein kinase (AMPK), and initiating a downstream metabolic cascade [30]. Previous studies have suggested that steviol glycosides may activate the CaMKKβ–AMPK pathway, thereby reducing fatty acid synthesis and sparing glycogen. This mechanism could partially explain the trends observed in pH decline and lipid content in SBE-treated broilers, although it remains to be directly verified in muscle tissue.

Amino acids and fatty acids serve as essential precursors for flavor formation in meat [31,32]. Saturated fatty acids (SFAs) are linked to an increased cardiovascular risk [33], while unsaturated fatty acids (UFA) (especially n–3 UFA) are anti-inflammatory and cardioprotective [34,35], and are major sources of volatile flavor compounds [36]. The activation of AMPK by steviol glycosides induces the phosphorylation of acetyl coenzyme A carboxylase, inhibiting fatty acid synthase (FAS) activity and suppressing lipogenesis. Concurrently, AMPK activation upregulates carnitine palmitoyltransferase 1 (CPT1) and enhances mitochondrial β–oxidation [37]. In this study, SBE supplementation resulted in a modest shift in its fatty acid profile: The proportion of polyunsaturated essential fatty acids (EFA) was slightly reduced (by −2–4 percentage points) in SBE groups. One possible explanation is that SBE’s antioxidant constituents (chlorogenic acids, etc.) protected some unsaturated fatty acids from oxidative loss during muscle storage, effectively concentrating non-EFAs in the lipid fraction. Another possibility is a metabolic effect altering fatty acid deposition. Importantly, the absolute changes in EFA content were small, and all meats remained good sources of EFAs. Significantly improved amino acid composition highlights the potential of SBE to modulate flavor precursors and nutrient density [31,38]. In SBE–300 broilers, the total free amino acid pool in breast muscle was –15% greater than in controls, with marked increases in umami-contributing amino acids such as glutamic acid and aspartic acid. This enrichment in umami amino acids provides a biochemical basis for the enhanced “broth freshness” flavor noted in sensory evaluation. On the other hand, bitter-tasting amino acids (e.g., valine, isoleucine) showed a smaller concurrent increase, which could introduce a slight bitter undertone. Overall, the net effect appears favorable to flavor, as the magnitude of umami enhancement outweighed the rise in bitter components.

Meat is highly perishable and spoilage is driven by microbial activity and endogenous enzymes leading to biochemical changes including TVB–N, which encompasses primary, secondary, and tertiary amines [39]. These changes are associated with undesirable flavors and potential toxicity [40]. Dietary supplementation with SBE effectively delayed the accumulation of TVB–N during storage, suggesting an improvement in shelf-life stability. Although TVB–N levels increased over time in all groups, the SBE–300 consistently maintained the lowest TVB–N level at all storage time points. Notably at 60 h, the TVB–N level was significantly lower than that of the Blank group and remained below the national limit (25 mg/100 g) (*p* < 0.05). These outcomes align with the antioxidant/antimicrobial effects reported for plant polyphenols; however, causal attribution cannot be established here because lipid oxidation and microbial loads were not directly quantified. Future work should integrate TBARS and microbial profiling to establish causal links. Polyphenols and flavonoids in plant extracts are known for scavenging free radicals, stabilizing membrane structure, inhibiting lipid oxidation, and limiting protein degradation [41,42]. Although no microbial counts or oxidative rancidity were measured in this study, the reduced TVB–N accumulation with SBE indicates a potential delay in spoilage progression.

In this study, 300 mg/kg emerged as an effective inclusion level that achieved most of the desirable improvements in meat quality. Higher supplementation (400 mg/kg) provided no further significant gains in key metrics (for instance, shear force and total amino acids were statistically similar between 300 and 400 mg groups), indicating diminishing returns beyond 300 mg/kg. Considering practical factors (feed additive cost and potential taste effects at high dosage), 300 mg/kg can be considered a suitable dose for SBE under our experimental conditions.

In the present meat-quality trial, 300 mg/kg SBE produced the most pronounced overall improvements among the tested doses, including lower shear force, more favorable a* values, a higher proportion of UAA and EAA, and slower TVB–N accumulation. Increasing the inclusion level to 400 mg/kg did not yield further significant gains in these traits, indicating diminishing returns for meat quality beyond 300 mg/kg. Thus, within the specific context of this experiment and when considering meat-quality traits alone, 300 mg/kg can be regarded as the most effective inclusion level among the tested doses. However, when our previous growth-performance results [12] are taken into account showing that 100–200 mg/kg improved body weight and average daily gain and feed efficiency, whereas 300 mg/kg impaired growth, 100–200 mg/kg remains the more appropriate supplementation range for routine commercial production. From a practical standpoint, 300 mg/kg SBE might be better positioned as a short-term finishing strategy in systems where maximizing meat quality is prioritized over maximal growth rate, and its economic viability should be evaluated case-by-case based on ingredient prices and market premiums for high-quality meat.

## 5. Conclusions

In this study, the effects of dietary SBE (0–400 mg/kg) on the meat quality of yellow-feathered broilers were systematically evaluated. The results showed that moderate SBE supplementation significantly improved sensory acceptability (broth freshness, tenderness), stabilized key physicochemical indices (higher a* and pH; lower shear force), and slowed freshness loss (lower TVB–N), SBE–00 performed best, with higher organoleptic scores, a more favorable amino-acid profile (higher UAA/EAA), moderated pH decline, and tighter TVB–N control. Taken together, an inclusion level of 300 mg/kg (SBE–300) produced the most pronounced improvements in meat-quality traits under the conditions of this experiment. Nevertheless, in light of our previous growth-performance study [12], which indicated that 100–200 mg/kg SBE improved growth and feed efficiency whereas 300 mg/kg reduced final body weight and average daily gain, 100–200 mg/kg should still be considered the preferred supplementation range for routine commercial production. Future studies should integrate growth performance, meat quality, and a full economic evaluation (including cost per kg of weight gain) within a single experimental design to refine dose recommendations for different production goals.

## Figures and Tables

**Table 1:** Basic feed composition and nutritional level.

Items	Composition, %	
	1–28 d	29–56 d
Ingredients		
Corn	49.60	54.27
Wheat middling and reddog	8.00	8.00
Rice bran	2.82	2.43
Oil	1.29	2.32
Soybean meal	26.73	19.03
Rapeseed meal	4.00	5.57
Cotton meal	3.00	4.00
L–Lys	0.10	0.16
DL–Met	0.19	0.17
CaHPO4	1.39	1.20
Limestone	1.50	1.50
Salt	0.30	0.30
Choline chloride	0.08	0.05
Premix1	1.00	1.00
Total	100.00	100.00
Nutrient level 2		
ME, Mcal/kg	2.80	2.90
Crude protein	20.01	18.08
Crude Fiber	3.52	3.41
Lys	1.09	0.98
Met	0.50	0.45
Met + Cys	0.85	0.79
Thr	0.76	0.68
Trp	0.24	0.21
Calcium	1.00	0.95
Total phosphorus	0.70	0.65
Available phosphorus	0.35	0.35

Note: (1) Premix provided per kilogram of diet: Vitamin A 12,000 IU; vitamin D3 2500 IU; vitamin E 20 mg; vitamin K3 3 mg; vitamin B1 3 mg; vitamin B2 8 mg; vitamin B6 7 mg; vitamin B12 0.03 mg; Pantothenic acid 20 mg; Nicotinic acid 50 mg; Biotin 0.05 B6 7 mg; vitamin B12 0.03 mg; Pantothenic acid 20 mg; Nicotinic acid 50 mg; Biotin 0.1 mg; Folic acid 1.5 mg; Cu 9 mg (25% anhydrous copper sulfate); Zn 110 mg (35% zinc sulfate monohydrate); Fe 100 mg (30% ferrous sulfate monohydrate); Mn 100 mg (31% Manganese sulfate monohydrate); Se 0.16 mg (1% sodium selenite); I 0.6 mg (1% calcium iodate). (2) Nutrient levels were calculated values.

**Table 2:** Chicken Meat Sensory Evaluation.

Item	1	2	3	4	5
Color (Visual inspection)	Muscle cross-section lacks luster, dull color	Muscle cross-section lacks luster, relatively dull color	Muscle cross-section has slight luster, relatively dull color	Muscle cross-section has luster, normal color	Muscle cross-section has rich luster, normal color
Juiciness (Visual inspection and chewing)	Extremely dry	Fairly dry	Moderate	Fairly juicy	Extremely juicy
Texture (Visual inspection)	Chicken breast meat is incomplete, with loose edges		Chicken breast meat is fairly complete, not loose		Chicken breast meat is complete, with tight tissue
Odor (Smelling the meat’s aroma)	Strong fishy or metallic odor	Natural aroma lost, with a strong fishy or metallic odor	Natural aroma faint, with a slight off-odor	Natural aroma, relatively fresh	Natural aroma, fresh
Umami (Smell and taste)	Extremely weak	Moderately weak	Moderate	Moderately strong	Extremely strong
Flavor (Taste with the tongue)	Extremely weak	Moderately weak	Moderate	Moderately strong	Extremely strong
Tenderness (Initial tooth chewing)	Extremely difficult	Moderately difficult	Moderate	Moderately easy	Extremely easy
Taste (Chewing)	Difficult to chew, dry and tough texture		Moderately easy to chew, slightly tough texture, slightly lacking in tenderness		Easy to chew, does not stick to teeth, tender and smooth meat texture

**Table 3:** Sensors used in PEN3 e-nose and their performance description.

Sensors	Responsive Substance
W1C	Sensitive to aromatic compounds
W5S	Sensitive to nitrogen oxides
W3C	Sensitive to ammonia and aromatic compounds
W6S	Sensitive to hydrogen
W5C	Sensitive to hydrocarbons, aromatic compounds
W1S	Sensitive to methane in the environment, with broad range
W1W	Sensitive to sulfur compounds, pyrazine, many terpenes, such as limonene
W2S	Sensitive to ethanol, some aromatic compounds, broad range
W2W	Sensitive to aromatic components, sulfur compounds
W3S	Sensitive to methane and some high concentration compounds

**Table 4:** Routine physical and chemical analysis of chicken breast in different concentration experimental groups (*n* = 5).

Item	Blank	SBE-50	SBE-100	SBE-200	SBE-300	SBE-400	SEM	*p*
Moisture content, %	70.33 ± 1.10	70.35 ± 0.99	70.94 ± 0.30	71.66 ± 2.10	71.76 ± 0.90	71.09 ± 0.66	0.218	0.248
Crude protein content, %	23.97 ± 0.97	24.02 ± 0.61	24.44 ± 0.31	24.49 ± 1.69	24.31 ± 0.83	25.16 ± 0.55	0.172	0.41
Ash content, %	1.40 ± 0.16	1.58 ± 0.22	1.65 ± 0.14	1.61 ± 0.34	1.61 ± 0.24	1.65 ± 0.26	0.042	0.587
Ether extract content, %	2.89 ± 0.22 ^d^	3.51 ± 0.37 ^ab^	3.84 ± 0.10 ^a^	3.00 ± 0.35 ^cd^	3.42 ± 0.09 ^b^	3.35 ± 0.40 ^bc^	0.075	<0.001
a*_45min_	3.28 ± 0.32 ^c^	3.57 ± 0.05 ^abc^	3.51 ± 0.25 ^bc^	3.50 ± 0.51 ^bc^	3.72 ± 0.22 ^ab^	3.94 ± 0.11 ^a^	0.061	0.027
b*_45min_	2.70 ± 0.15	2.70 ± 0.26	2.78 ± 0.50	2.75 ± 0.20	2.81 ± 0.50	2.88 ± 0.34	0.06	0.961
L*_45min_	46.42 ± 0.35 ^a^	46.39 ± 0.97 ^a^	46.38 ± 0.24 ^a^	45.70 ± 1.15 ^ab^	44.99 ± 0.85 ^b^	43.98 ± 0.46 ^c^	0.209	<0.001
a*_24h_	2.71 ± 0.11 ^b^	2.81 ± 0.41 ^ab^	2.92 ± 0.07 ^ab^	2.91 ± 0.14 ^ab^	3.02 ± 0.35 ^ab^	3.20 ± 0.40 ^a^	0.055	0.15
b*_24h_	2.42 ± 0.24 ^c^	2.63 ± 0.23 ^c^	3.16 ± 0.28 ^b^	3.72 ± 0.11 ^a^	3.73 ± 0.22 ^a^	3.32 ± 0.13 ^b^	0.099	<0.001
L*_24h_	54.38 ± 1.01 ^c^	55.22 ± 0.42 ^b^	55.58 ± 0.21 ^b^	55.64 ± 0.33 ^b^	56.71 ± 0.20 ^a^	56.65 ± 0.11 ^a^	0.17	<0.001
pH_45min_	6.02 ± 0.04 ^e^	6.03 ± 0.13 ^e^	6.19 ± 0.05 ^d^	6.31 ± 0.08 ^bc^	6.40 ± 0.07 ^ab^	6.46 ± 0.14 ^a^	0.035	<0.001
pH_24h_	5.35 ± 0.04 ^e^	5.44 ± 0.01 ^d^	5.50 ± 0.03 ^c^	5.49 ± 0.01 ^c^	5.56 ± 0.03 ^b^	5.64 ± 0.01 ^a^	0.017	<0.001
Water loss, %	16.18 ± 0.34	16.34 ± 0.26	16.49 ± 0.39	16.51 ± 0.43	16.59 ± 0.33	16.67 ± 0.38	0.067	0.336
Drip loss, %	25.12 ± 0.71	25.58 ± 0.18	25.61 ± 0.39	25.65 ± 0.47	25.59 ± 0.59	25.69 ± 0.48	0.09	0.52
Shear force, kgf	3.98 ± 0.31 ^a^	3.13 ± 0.52 ^b^	3.34 ± 0.19 ^b^	3.37 ± 0.37 ^b^	3.36 ± 0.43 ^b^	3.37 ± 0.18 ^b^	0.076	0.021
Storage loss, %	7.13 ± 0.64 ^a^	6.33 ± 1.15 ^ab^	6.30 ± 0.64 ^ab^	5.38 ± 0.58 ^bc^	5.33 ± 016 ^bc^	4.96 ± 0.26 ^c^	0.187	0.001

Note: Different letters above the bars indicate significant differences among dietary treatments (*p* < 0.05).

**Table 5:** Routine physical and chemical analysis of chicken thigh in different concentration experimental groups (*n* = 5).

Item	Blank	SBE-50	SBE-100	SBE-200	SBE-300	SBE-400	SEM	*p*
Moisture content, %	72.40 ± 0.81 ^b^	73.67 ± 0.33 ^a^	72.32 ± 1.72 ^b^	74.04 ± 0.59 ^a^	74.09 ± 0.43 ^a^	72.21 ± 0.64 ^b^	0.212	0.002
Crude protein content, %	21.38 ± 0.65 ^bc^	20.98 ± 0.29 ^bc^	22.05 ± 1.59 ^b^	20.55 ± 0.51 ^c^	21.77 ± 0.32 ^b^	23.56 ± 0.50 ^a^	0.22	<0.001
Ash content, %	1.23 ± 0.06 ^b^	1.22 ± 0.10 ^b^	1.30 ± 0.32 ^ab^	1.38 ± 0.14 ^ab^	1.43 ± 0.26 ^ab^	1.56 ± 0.24 ^a^	0.041	0.123
Ether extract content, %	5.84 ± 0.30 ^a^	4.69 ± 0.09 ^c^	4.82 ± 0.38 ^c^	5.25 ± 0.18 ^b^	3.20 ± 0.14 ^e^	3.79 ± 0.33 ^d^	0.169	<0.001
a*_45min_	5.38 ± 1.05	5.73 ± 0.43	5.89 ± 0.63	5.84 ± 0.54	5.88 ± 0.31	5.87 ± 0.29	0.105	0.735
b*_45min_	2.91 ± 0.63	2.94 ± 0.36	2.96 ± 0.33	3.07 ± 0.43	3.06 ± 0.35	3.09 ± 0.28	0.07	0.972
L*_45min_	48.60 ± 0.82 ^a^	48.64 ± 0.30 ^a^	46.62 ± 0.44 ^b^	46.39 ± 0.16 ^b^	46.22 ± 0.30 ^b^	46.57 ± 0.44 ^b^	0.206	<0.001
a*_24h_	2.74 ± 0.12	2.96 ± 0.13	2.97 ± 0.26	3.00 ± 0.35	3.04 ± 0.30	3.05 ± 0.46	0.053	0.596
b*_24h_	3.38 ± 0.32 ^b^	3.60 ± 0.24 ^ab^	3.61 ± 0.19 ^ab^	3.66 ± 0.26 ^ab^	3.64 ± 0.18 ^ab^	3.91 ± 0.21 ^a^	0.049	0.058
L*_24h_	54.19 ± 0.34 ^b^	55.67 ± 0.24 ^a^	55.59 ± 0.16 ^a^	55.63 ± 0.27	55.65 ± 0.53 ^a^	55.67 ± 0.17 ^a^	0.113	<0.001
pH_45min_	6.31 ± 0.10 ^b^	6.33 ± 0.06 ^b^	6.42 ± 0.04 ^a^	6.47 ± 0.04 ^a^	6.48 ± 0.07 ^a^	6.50 ± 0.06 ^a^	0.018	<0.001
pH_24h_	5.80 ± 0.02 ^d^	5.80 ± 0.01 ^d^	5.86 ± 0.01 ^cd^	5.91 ± 0.09 ^bc^	5.94 ± 0.04 ^b^	6.03 ± 0.05 ^a^	0.017	<0.001
Water loss, %	16.29 ± 0.62	16.32 ± 0.38	16.30 ± 0.12	16.37 ± 0.26	16.37 ± 0.78	16.34 ± 0.49	0.082	1
Drip loss, %	29.00 ± 1.52 ^b^	30.85 ± 0.48 ^a^	30.85 ± 0.32 ^a^	30.89 ± 0.52 ^a^	30.91 ± 0.14 ^a^	30.89 ± 0.15 ^a^	0.175	<0.001
Shear force, kgf	4.64 ± 0.29	4.64 ± 0.14	4.70 ± 0.29	4.72 ± 0.29	4.78 ± 0.27	4.74 ± 0.38	0.049	0.965
Storage loss, %	2.66 ± 0.38	2.50 ± 0.41	2.47 ± 0.27	2.46 ± 0.25	2.45 ± 0.26	2.46 ± 0.13	0.051	0.871

Note: Different letters above the bars indicate significant differences among dietary treatments (*p* < 0.05).

## Data Availability

The data presented in this study are available on request from the corresponding author.

## Supplementary Materials

The following supporting information can be downloaded at: https://www.mdpi.com/article/10.3390/ani16071026/s1, Table S1: Sensory Evaluation; Table S2: E-nose Measurement; Table S3: TVB–N content; Table S4: Fatty acid proportions; Table S5: Amino acid contents.
