# Supplementary material for: Effects of Supplementing Stevia rebaudiana Bertoni Extract in the Diet of Yellow-Feathered Broilers on Meat Quality
Source: Animals (Basel). 2026 Mar 27;16(7):1026. doi: 10.3390/ani16071026 (PMC13072137; doi:10.3390/ani16071026)
Supplement: Supplementary file 1 [file animals-16-01026-s001.zip › animals-4167681-supplementary.pdf]

Supplementary Table S1. Sensory Evaluation.

Sample	Blank	SBE-50	SBE-100	SBE-200	SBE-300	SBE-400	SEM	P-value
Color	4.67±0.52	4.5±0.55	4.33±0.52	4.17±0.41	4.33±0.52	4.17±0.75	0.09	0.596
Juiciness	3.5±0.55	3.67±0.52	3.67±0.52	3.5±0.55	3.67±0.52	3.67±0.52	0.082	0.975
Texture	4.67±0.52	4.5±0.55	4.5±0.55	4.5±0.55	4.5±0.55	4.5±0.55	0.084	0.992
Odor	3.5±0.55	4±0.63^{a}	3.67±0.52	3.17±0.41^{b}	3.5±0.55	3.67±0.52	0.092	0.194
Umami	3.33±0.52^{c}	4.5±0.55^{a}	3.83±0.41^{bc}	3.83±0.41^{bc}	4.5±0.55^{a}	4.33±0.52^{ab}	0.105	0.001
Flavor	3.5±0.55	3.5±0.55	3.33±0.52	3.67±0.52	3.5±0.55	3.67±0.52	0.084	0.889
Tenderness	3.33±0.52	3±0^{b}	3.83±0.41^{a}	3.33±0.52	3.5±0.55	3.67±0.52^{a}	0.084	0.059
Taste	3.5±0.55	3.33±0.52	3.67±0.82	3.33±0.52	3.67±0.52	3.33±0.52	0.093	0.792

Supplementary Table S2. E-nose Measurement

Class	Sample	Blank	SBE-50	SBE-100	SBE-200	SBE-300	SBE-400	SEM	P-value
chicken thigh	W1C	1.031±0.031^{b}	1.075±0.01^{a}	1.074±0.006^{a}	1.07±0.008^{a}	1.068±0.009^{a}	1.043±0.004^{b}	0.002	＜0.001
	W5S	1.808±0.279^{b}	1.182±0.145^{c}	1.147±0.046^{c}	1.23±0.095^{c}	1.269±0.106^{c}	2.279±0.376^{a}	0.085	＜0.001
	W3C	1.053±0.019^{b}	1.075±0.006^{a}	1.082±0.003^{a}	1.08±0.004^{a}	1.076±0.005^{a}	1.056±0.002^{b}	0.003	＜0.001
	W6S	1±0.001^{c}	1.002±0.003^{bc}	1.004±0.003^{ab}	1.005±0.003^{ab}	1.005±0.002^{ab}	1.007±0.003^{a}	0.001	0.012
	W5C	1.047±0.014^{c}	1.072±0.001^{a}	1.076±0.003^{a}	1.073±0.003^{a}	1.07±0.005^{a}	1.057±0.001^{b}	0.002	＜0.001
	W1S	1.987±0.384^{b}	1.118±0.084^{c}	1.089±0.026^{c}	1.13±0.061^{c}	1.149±0.057^{c}	3.607±0.773^{a}	0.18	＜0.001
	W1W	1.014±0.008^{b}	1.007±0.004^{b}	1.007±0.002^{b}	1.01±0.004^{b}	1.012±0.004^{b}	1.046±0.021^{a}	0.003	＜0.001
	W2S	4.16±0.177^{b}	4.592±0.316^{ab}	4.566±0.209^{ab}	4.889±0.603^{a}	4.539±0.145^{ab}	4.736±0.34^{a}	0.069	0.049
chicken breast	W1C	1.054±0.008^{b}	1.057±0.009^{b}	1.068±0.002^{a}	1.074±0.007^{a}	1.071±0.011^{a}	1.078±0.001^{a}	0.002	＜0.001
	W5S	2.101±0.695^{a}	1.865±0.592^{a}	1.262±0.062^{b}	1.173±0.066^{b}	1.298±0.189^{b}	1.095±0.032^{b}	0.094	＜0.001
	W3C	1.06±0.004^{b}	1.063±0.009^{b}	1.077±0.006^{a}	1.076±0.003^{a}	1.078±0.005^{a}	1.081±0.002^{a}	0.002	＜0.001
	W6S	1.008±0.007	1.006±0.004	1.007±0.003	1.005±0.003	1.008±0.007	1.002±0.001	0.001	0.466
	W5C	1.061±0.004^{b}	1.064±0.007^{b}	1.077±0.001^{a}	1.075±0.002^{a}	1.077±0.003^{a}	1.075±0.003^{a}	0.001	＜0.001
	W1S	2.722±1.192^{a}	2.393±1.048^{a}	1.165±0.029^{b}	1.114±0.035^{b}	1.187±0.135^{b}	1.073±0.011^{b}	0.167	＜0.001
	W1W	1.043±0.035^{ab}	1.031±0.026^{a}	1.017±0.003^{b}	1.012±0.004^{b}	1.015±0.01^{b}	1.009±0.002^{b}	0.004	0.029
	W2S	4.991±1.112^{b}	4.883±1.024^{b}	4.737±0.433^{a}	4.644±0.272^{a}	4.628±0.506^{a}	4.427±0.13^{a}	0.118	0.731

Supplementary Table S3. TVB-N content

Class	Sample	Blank	SBE-50	SBE-100	SBE-200	SBE-300	SBE-400	SEM	P-value
chicken thigh	0h	5.41±0.28	5.28±0.32	5.23±0.38	5.05±0.52	5.17±0.34	5.15±0.09	0.056	0.595
	12h	5.57±0.25	5.46±0.38	5.41±0.38	5.23±0.54	5.33±0.3	5.31±0.08	0.057	0.608
	24h	5.75±0.28	5.65±0.41	5.57±0.36	5.39±0.6	5.53±0.32	5.47±0.07	0.061	0.626
	36h	9.26±0.16^{a}	8.95±0.07^{b}	8.56±0.07^{c}	8.55±0.08^{c}	8.15±0.14^{d}	8.26±0.12^{d}	0.067	＜0.001
	48h	15.92±0.21^{a}	15.04±0.24^{b}	13.9±0.16^{c}	14.01±0.18^{c}	12.82±0.13^{d}	12.96±0.32^{d}	0.188	＜0.001
	60h	27.43±0.41^{a}	25.18±0.34^{b}	22.51±0.32^{c}	22.8±0.26^{c}	20.08±0.17^{d}	20.36±0.32^{d}	0.44	＜0.001
	72h	67.65±1.04^{a}	59.05±0.95^{b}	50.86±1.13^{c}	50.74±0.74^{c}	42.76±0.77^{d}	43.79±0.59^{d}	1.468	＜0.001
chicken breast	0h	6.14±0.06	6.16±0.1	6.15±0.18	6.15±0.09	6.16±0.12	6.15±0.09	0.017	0.999
	12h	6.35±0.07	6.33±0.12	6.33±0.19	6.32±0.08	6.32±0.18	6.32±0.15	0.022	1.000
	24h	6.6±0.11	6.52±0.11	6.54±0.23	6.53±0.12	6.52±0.13	6.49±0.27	0.027	0.936
	36h	11.07±0.11^{a}	10.62±0.13^{b}	10.22±0.09^{c}	10.15±0.14^{c}	9.78±0.09^{d}	9.81±0.11^{d}	0.078	＜0.001
	48h	19.11±0.21^{a}	17.76±0.2^{b}	16.53±0.22^{c}	16.48±0.27^{c}	15.39±0.21^{d}	15.5±0.12^{d}	0.222	＜0.001
	60h	32.97±0.4^{a}	29.65±0.37^{b}	26.93±0.33^{c}	26.65±0.45^{c}	24.25±0.38^{d}	24.38±0.1^{d}	0.519	＜0.001
	72h	80.71±1.75^{a}	69.44±0.83^{b}	60.85±1.16^{c}	59.41±1.38^{d}	52.32±1.28^{e}	52.56±0.35^{e}	1.692	＜0.001

Supplementary Table S4. Fatty acid proportions

Class	Sample	Blank	SBE-50	SBE-100	SBE-200	SBE-300	SBE-400	SEM	P-value
chicken thigh	C14:0	0.35±0.03^{c}	0.48±0.02^{b}	0.49±0.03^{b}	0.50±0.03^{ab}	0.48±0.02^{b}	0.52±0.02^{a}	0.01	<0.001
	C16:0	24.08±0.65^{b}	26.92±0.92^{a}	26.47±1.32^{a}	26.80±1.00^{a}	26.01±0.35^{a}	25.01±0.36	0.216	<0.001
	C16:1	2.97±0.17^{d}	4.95±0.18^{c}	4.74±0.23^{c}	5.63±0.19^{a}	5.26±0.10^{c}	4.79±0.22^{c}	0.145	<0.001
	C18:0	11.61±0.47^{d}	10.13±0.32^{c}	10.43±0.77^{c}	8.86±0.30^{b}	9.45±0.16^{a}	8.80±0.25^{b}	0.178	<0.001
	C18:1n9C	24.77±0.84^{c}	32.16±1.08^{b}	32.25±1.32^{b}	34.57±1.04^{a}	31.89±0.38^{b}	32.04±0.56^{b}	0.535	<0.001
	C18:2n6C	19.38±0.51^{d}	18.85±0.62^{cd}	18.64±0.91^{c}	17.20±0.55^{b}	17.37±0.27^{ab}	17.96±0.27^{ab}	0.16	<0.001
	C20:1n9	0.19±0.01^{d}	0.26±0.01^{c}	0.26±0.01^{bc}	0.25±0.01^{b}	0.23±0.01^{a}	0.26±0.02^{bc}	0.004	<0.001
	C18:3n3	0.37±0.03^{d}	0.53±0.02^{c}	0.52±0.03^{cb}	0.53±0.02^{cb}	0.50±0.02^{b}	0.59±0.02^{a}	0.012	<0.001
	C20:2	0.39±0.03^{c}	0.27±0.02^{ab}	0.29±0.02^{b}	0.24±0.07^{a}	0.24±0.01^{a}	0.26±0.02^{bc}	0.01	<0.001
	C20:3n6	1.17±0.08^{a}	0.97±0.03^{bc}	1.00±0.11^{b}	0.85±0.04^{d}	0.92±0.03^{cd}	0.73±0.02^{e}	0.025	<0.001
	C20:4	9.33±0.49^{a}	6.70±0.20^{b}	6.89±0.70^{b}	5.00±0.12^{c}	4.85±0.13^{c}	5.51±0.12^{d}	0.265	<0.001
	C20:5n3	2.37±0.10^{a}	1.64±0.05^{b}	1.70±0.18^{b}	1.14±0.04^{c}	1.24±0.03^{c}	1.42±0.05^{d}	0.07	<0.001
	C22:6n3	6.14±0.30^{a}	4.81±0.18^{b}	5.10±0.52^{b}	3.67±0.20^{c}	3.95±0.08^{c}	3.62±0.14^{c}	0.158	<0.001
	SFA	36.04±1.00^{a}	37.53±1.25^{a}	37.39±2.06^{a}	36.16±1.33^{a}	35.95±0.47^{a}	34.33±0.52^{b}	0.261	0.001
	UFA	67.10±1.61^{b}	71.16±2.32^{a}	71.39±3.44^{a}	69.07±2.09^{ab}	66.44±0.80^{b}	67.18±1.25^{b}	0.465	<0.001
	EFA	37.60±1.35^{a}	32.54±1.03^{b}	32.85±2.10^{b}	27.54±0.87^{cd}	27.91±0.39^{cd}	29.11±0.56^{cd}	0.622	<0.001

Class	Sample	Blank	SBE-50	SBE-100	SBE-200	SBE-300	SBE-400	SEM	P-value
chicken breast	C14:0	0.63±0.03[b]	0.67±0.04[b]	0.67±0.03[b]	0.70±0.03[a]	0.68±0.03[a]	0.61±0.04[b]	0.007	<0.001
	C16:0	25.17±1.10[c]	28.23±1.66 [ab]	29.37±1.10[a]	29.05±1.16[ab]	27.59±1.27[a]	25.55±1.42[b]	0.339	<0.001
	C16:1	6.86±0.32[c]	8.17±0.50[ab]	7.74±0.32[b]	8.36±0.34[a]	8.44±0.39[a]	6.14±0.34[d]	0.154	<0.001
	C18:0	6.99±0.31[ab]	7.44±0.42[ab]	7.26±0.81[ab]	6.09±2.85[b]	7.75±0.28[a]	8.24±0.38[a]	0.222	0.098
	C18:1n9C	38.37±1.65[b]	41.21±2.48[a]	41.18±1.54[a]	41.75±1.72[a]	36.40±1.60[b]	36.36±1.83[b]	0.477	<0.001
	C18:2n6C	21.83±0.92[a]	19.01±1.11[bc]	19.13±0.74[bc]	19.60±0.79[bc]	19.14±0.87[bc]	20.24±1.06[b]	0.219	<0.001
	C20:1n9	0.35±0.02[c]	0.39±0.03[a]	0.35±0.02[c]	0.35±0.02[c]	0.32±0.01[c]	0.34±0.01[bc]	0.005	<0.001
	C18:3n3	0.87±0.03[a]	0.74±0.05[b]	0.75±0.03[b]	0.81±0.03[a]	0.74±0.03[b]	0.73±0.04[b]	0.01	<0.001
	C20:2	0.20±0.01[a]	0.16±0.01[b]	0.14±0.01[c]	0.15±0.01[c]	0.17±0.01[b]	0.19±0.01[a]	0.004	<0.001
	C20:3n6	0.31±0.01[c]	0.33±0.02[c]	0.32±0.01[c]	0.36±0.01[b]	0.47±0.02[a]	0.43±0.02[a]	0.011	<0.001
	C20:4	2.77±0.13[c]	2.42±0.14[e]	2.10±0.10[f]	2.58±0.09[d]	3.12±0.13[b]	3.76±0.18[a]	0.092	<0.001
	C20:5n3	0.68±0.03[d]	0.66±0.04[d]	0.56±0.02[c]	0.66±0.02[d]	0.82±0.03[b]	0.96±0.05[a]	0.023	<0.001
	C22:6n3	1.82±0.16[c]	1.95±0.14[c]	1.64±0.06[b]	1.89±0.08[c]	2.63±0.14[a]	2.66±0.16[a]	0.071	<0.001
	SFA	32.79±1.42[c]	36.34±2.12[ab]	37.30±1.88[a]	35.85±2.32[ab]	36.02±1.57[ab]	34.40±1.83[bc]	0.382	0.004
	UFA	74.07±3.21[ab]	75.05±4.50[ab]	73.90±2.80[ab]	76.51±3.11[a]	72.27±3.09[ab]	71.81±3.55[b]	0.592	0.202
	EFA	27.97±1.21[a]	24.78±1.47[b]	24.18±0.92[b]	25.54±1.00[bc]	26.46±1.07[c]	28.34±1.35[a]	0.319	<0.001

Supplementary Table S5.Amino acid contents

Class	Sample	Blank	SBE-50	SBE-100	SBE-200	SBE-300	SBE-400	SEM	P-value
chicken thigh	Asp	768.15±16.10	771.35±39.86	772.92±36.43	754.48±22.49	787.05±29.78	751.99±10.88	4.752	0.296
	Clu	1261.24±37.25^{ab}	1260.86±63.83^{ab}	1255.53±51.97^{b}	1241.35±30.33^{b}	1314.59±51.35^{a}	1264.18±14.47^{ab}	7.89	0.126
	Thr	315.08±8.10^{a}	323.07±17.33^{a}	314.06±12.18^{a}	288.64±15.42^{b}	307.93±13.15^{a}	292.52±5.33^{b}	2.858	<0.001
	Ser	318.92±4.99^{a}	325.04±15.98^{a}	325.47±15.13^{a}	289.63±12.28^{b}	327.29±14.04^{a}	238.72±9.53^{c}	5.743	<0.001
	Gly	355.28±9.60^{a}	344.99±17.23^{b}	349.75±18.77^{b}	345.97±16.42^{b}	372.24±21.00^{a}	342.37±5.35^{b}	2.958	0.028
	Ala	365.22±7.39^{ab}	371.49±18.85^{a}	363.16±16.65^{ab}	352.51±11.71^{b}	379.69±13.60^{a}	319.80±7.17^{c}	3.843	<0.001
	Pro	513.53±9.37^{a}	514.12±27.28^{a}	505.09±16.66^{a}	447.74±12.76^{b}	524.62±17.98^{a}	455.18±15.68^{b}	5.784	<0.001
	Val	483.47±9.25^{a}	479.81±23.95^{b}	476.67±16.45^{b}	521.91±18.80^{a}	532.73±24.54^{a}	465.55±8.21^{b}	5.036	<0.001
	Met	263.22±5.16^{a}	264.38±13.65^{a}	263.87±10.68^{a}	233.26±9.37^{b}	254.85±9.93^{a}	213.12±3.20^{c}	3.562	<0.001
	Ile	384.81±9.53^{ab}	371.35±22.27^{b}	386.71±14.89^{b}	387.42±11.42^{b}	428.12±13.26^{a}	372.99±2.34^{b}	3.814	<0.001
	Leu	233.34±6.70^{a}	229.29±13.18^{a}	229.01±14.47^{a}	204.82±8.26^{b}	229.40±8.49^{a}	187.91±3.05^{c}	3.214	<0.001
	Tyr	307.16±6.52^{a}	307.67±15.76^{a}	314.28±14.02^{a}	281.13±9.66^{b}	313.28±10.70^{a}	268.42±1.40^{b}	3.39	<0.001

Class	Sample	Blank	SBE-50	SBE-100	SBE-200	SBE-300	SBE-400	SEM	P-value
	Phe	398.56±9.57^{bc}	386.27±21.90^{cd}	403.69±16.47^{bc}	407.89±13.68^{b}	440.19±16.77^{a}	375.44±2.59^{d}	4.117	<0.001
	Lys	643.88±13.80^{c}	639.02±32.85^{c}	646.60±26.15^{c}	673.18±20.44^{b}	736.48±24.02^{a}	643.09±5.39^{c}	6.756	<0.001
	His	633.23±21.10^{c}	628.85±30.12^{c}	633.36±53.15^{c}	760.83±25.34^{b}	837.13±27.42^{a}	531.72±42.18^{d}	17.703	<0.001
	Arg	493.30±13.85^{a}	486.80±23.75^{ab}	465.03±22.25^{b}	330.60±16.31^{d}	388.75±15.93^{c}	483.23±20.96^{ab}	10.68	<0.001
	UAA	2029.38±53.13^{ab}	2032.21±103.67^{ab}	2028.45±87.78^{ab}	1995.83±52.75^{b}	2101.65±80.73^{a}	2016.17±25.32^{ab}	12.425	0.226
	BAA	3696.65±83.71^{b}	3665.99±189.47^{b}	3708.07±173.07^{b}	3685.90±119.36^{b}	4091.37±141.39^{a}	3286.58±79.23^{c}	44.683	<0.001
	SAA	2012.34±39.47^{a}	2006.17±100.28^{a}	1968.67±76.86^{ab}	1839.63±68.68^{c}	1981.34±78.49^{ab}	1903.48±35.00^{bc}	15.021	0.001
	AAA	570.38±11.63^{a}	572.04±29.34^{a}	578.16±24.61^{a}	514.38±19.00^{b}	568.13±20.56^{a}	481.540±4.33^{c}	6.881	<0.001
	EAA	2966.20±71.90^{b}	2933.95±151.79^{b}	2976.80±151.85^{b}	3067.78±99.59^{b}	3364.30±113.39^{a}	2699.36±61.01^{c}	37.704	<0.001
	Asp	642.53±20.20^{c}	679.86±22.71^{b}	652.28±20.94^{c}	638.10±16.65^{c}	706.21±16.02^{a}	709.79±11.94^{a}	5.676	<0.001
chicken breast	Clu	1162.62±34.37^{bc}	1182.45±43.17^{b}	1137.92±26.83^{c}	1169.26±31.31^{bc}	1267.92±32.98^{a}	1287.18±25.54^{a}	10.716	<0.001

Class	Sample	Blank	SBE-50	SBE-100	SBE-200	SBE-300	SBE-400	SEM	P-value
	Thr	263.61±9.01[c]	295.68±11.33[a]	278.49±14.57[b]	263.05±6.71[c]	284.09±6.73[ab]	291.89±4.57[a]	2.586	<0.001
	Ser	163.83±7.53[d]	214.99±7.37[a]	193.03±21.89[b]	127.60±2.96[e]	192.62±4.06[b]	178.75±4.35[c]	4.917	<0.001
	Gly	402.10±9.73[a]	363.60±16.97[b]	366.48±16.17[b]	393.80±11.34[a]	400.35±9.98[a]	407.80±7.66[a]	3.523	<0.001
	Ala	300.26±10.57[a]	330.09±11.06[b]	305.43±19.97[a]	261.03±9.11[c]	327.04±6.77[b]	328.71±5.49[b]	4.477	<0.001
	Pro	516.38±16.78[a]	478..95±17.80[b]	464.22±23.77[b]	444.70±11.07[a]	525.55±53.21[a]	457.73±68.70[a]	7.790	0.004
	Val	520.74±14.29[b]	450.48±49.87[c]	465.80±28.08[c]	500.75±11.42[b]	552.55±12.42[a]	560.76±8.92[a]	7.951	<0.001
	Met	189.88±8.31[c]	237.31±8.14[a]	218.49±25.99[b]	174.08±6.79[d]	208.43±4.49[b]	208.02±4.56[b]	3.887	<0.001
	Ile	301.46±10.91[c]	318.87±10.01[ab]	313.90±15.22[b]	295.37±9.28[c]	329.48±7.71[a]	324.96±7.05[ab]	2.606	<0.001

Class	Sample	Blank	SBE-50	SBE-100	SBE-200	SBE-300	SBE-400	SEM	P-value
	Leu	169.14±5.71^{c}	207.08±9.07^{a}	189.43±20.65^{b}	157.68±4.62^{c}	194.58±5.92^{ab}	197.39±4.26^{ab}	3.280	<0.001
	Tyr	233.67±9.12^{cd}	281.23±8.93^{a}	264.74±23.47^{b}	225.91±7.23^{d}	245.38±5.41^{c}	236.44±8.31^{cd}	3.741	<0.001
	Phe	326.75±12.14^{c}	340.91±10.39^{c}	332.41±18.59^{c}	306.41±9.51^{c}	365.47±9.63^{a}	367.79±8.34^{a}	4.080	<0.001
	Lys	560.93±19.49^{bc}	567.77±18.47^{b}	552.11±18.34^{bc}	545.03±16.27^{c}	609.18±11.91^{a}	606.72±11.06^{a}	4.956	<0.001
	His	646.10±27.49^{b}	559.61±23.83^{c}	500.66±22.98^{d}	431.66±15.08^{e}	720.76±13.11^{a}	713.42±13.39^{a}	18.427	<0.001
	Arg	376.08±12.93^{c}	497.29±27.83^{b}	503.08±28.35^{b}	536.28±14.39^{a}	389.24±18.39^{c}	399.96±10.96^{c}	11.163	<0.001
	UAA	1805.14±54.44^{bc}	1862.32±65.47^{b}	1790.20±46.77^{c}	1807.35±47.94^{bc}	1974.14±48.76^{a}	1996.97±37.45^{a}	16.097	<0.001
	BAA	3108.15±115.56^{cd}	3206.71±108.67^{bc}	3028.99±185.69^{d}	2708.43±78.80^{e}	3391.45±84.79^{a}	3291.22±93.95^{ab}	41.203	<0.001

Class	Sample	Blank	SBE-50	SBE-100	SBE-200	SBE-300	SBE-400	SEM	P-value
	SAA	1862.79±48.80^{b}	1937.13±95.99^{ab}	1919.27±89.28^{ab}	1954.90±37.89^{a}	1953.27±51.85^{a}	1989.12±26.92^{a}	11.845	0.040
	AAA	423.55±17.38^{de}	518.54±17.03^{a}	483.23±49.36^{b}	399.99±13.97^{e}	453.82±9.57^{c}	444.46±12.27^{cd}	7.509	<0.001
	EAA	2538.31±93.80^{bc}	2605.56±87.41^{b}	2458.68±135.49^{c}	2223.08±66.85^{d}	2791.88±58.95^{a}	2775.43±53.89^{a}	35.481	<0.001
